# Three years of clinical experience with a genome-wide cfDNA screening test for aneuploidies and copy-number variants

**DOI:** 10.1038/s41436-021-01135-8

**Published:** 2021-03-17

**Authors:** Erica Soster, Theresa Boomer, Susan Hicks, Samantha Caldwell, Brittany Dyr, Jason Chibuk, Eyad Almasri

**Affiliations:** Integrated Genetics, San Diego, CA USA

## Abstract

**Purpose:**

Pregnant women have unprecedented choices for prenatal screening and testing. Cell-free DNA (cfDNA) offers the option to screen for aneuploidy of all chromosomes and genome-wide copy-number variants (CNVs), expanding screening beyond the common trisomies (“traditional” cfDNA). We sought to review the utilization trends and clinical performance characteristics of a commercially available genome-wide cfDNA test, with a subset having available diagnostic testing outcomes.

**Methods:**

Retrospective analysis of 55,517 samples submitted for genome-wide cfDNA screening at a commercial laboratory, assessing indications, demographics, results, and performance. The cohort was broken into three “testing years”’ to compare trends.

**Results:**

Indications shifted over time, with a decrease in referrals for ultrasound findings (22.0% to 12.0%) and an increase in no known high-risk indication (3.0% to 16.6%). Of the positive results, 25% would be missed with traditional cfDNA screening. High sensitivity and specificity were observed with a positive predictive value (PPV) of 72.6% for genome-wide CNVs and 22.4% for rare autosomal trisomies (RATs).

**Conclusion:**

A broader patient population is utilizing genome-wide cfDNA, yet positivity rates and the contribution of genome-wide events have remained stable at approximately 5% and 25%, respectively. Test performance in a real-world clinical population shows high PPVs in those CNVs tested, with diagnostic outcomes in over 40% of positive cases.

## INTRODUCTION

The recent decade has seen rapid advances in prenatal genetic technologies, providing pregnant women unprecedented screening and diagnostic testing options. Cell-free DNA (cfDNA) screening for trisomy 21 offered women an option to screen for Down syndrome with a higher sensitivity and specificity than conventional screening methods.^1^ Since becoming commercially available in 2011, “traditional” cfDNA rapidly expanded to include trisomies 18 and 13, fetal sex, and sex chromosome aneuploidies. Select common microdeletions and additional aneuploidies are also available as optional content from some laboratories.^2^ Due to the screening nature of the testing, diagnostic confirmation is recommended for all positive results.

Even with the enhanced sensitivity of cfDNA over traditional options, screening for trisomies 21, 18, 13 (“common trisomies”) and sex chromosome aneuploidies (SCAs) only detects between 75% and 83% of chromosome anomalies identifiable by fetal karyotype, leaving approximately 17% and 25% of affected pregnancies undetectable by cfDNA.^3,4^ Genome-wide cfDNA screens the entire chromosome complement for well-defined, recurrent conditions (such as trisomy 21) as well as unique, de novo copy-number variants (CNVs), allowing detection of more genetic abnormalities while maintaining high specificity.^5,6^

A commercially available genome-wide cfDNA test launched in 2015, allowing for screening of common trisomies and SCAs, autosomal aneuploidies, CNVs 7 megabases (Mb) or larger, and a select list of microdeletions below 7 Mb.^5^ Similar tests screening for genome-wide events are now offered by multiple clinical laboratories, including in Asia and Europe. Early data from the second phase of the TRIDENT study in the Netherlands shows that following a 30-minute pretest counseling session with a certified provider, 78% of women opted for a genome-wide approach over a traditional cfDNA approach.^7^ Professional societies do not recommend genome-wide cfDNA screening citing a need for more published data on test performance and clinical validity.^8,9^ A previous study outlined the demographic and ordering trends of the first 10,000 genome-wide cfDNA tests, but did not have any data on diagnostic testing outcomes.^10^ The aim of this study was to provide additional published data on genome-wide cfDNA performance, utilization, and diagnostic testing outcomes.

The current study details three years of clinical experience screening over 55,000 clinical samples, including demographics and testing indications, from a commercially available genome-wide cfDNA test. Additionally, diagnostic outcomes were available for a portion of these samples, including 42.5% of screen-positive results, allowing performance calculations for this subset.

## MATERIALS AND METHODS

### Study cohort

A retrospective analysis was performed on 55,517 consecutive maternal blood samples from singleton pregnancies submitted for clinical genome-wide cfDNA screening at a commercial laboratory (CLIA-certified, College of American Pathologists–accredited, and ISO-accredited) from 31 August 2015 to 31 August 2018. Data were compiled and analyzed overall as well as in year-over-year cohorts based on accession date: 31 August 2015 to 31 August 2016 as year 1, 1 September 2016 to 31 August 2017 as year 2, and 1 September 2017 to 31 August 2018 as year 3. Demographic information was collected and analyzed where available. Gestational age was determined by last menstrual period or ultrasound as provided on the test requisition form (TRF) by the ordering clinician. Maternal weight is not required for testing and as such, was not available for all specimens. Cases with maternal weight below 80 pounds (*n* = 23) were excluded as outliers likely related to error on the TRF or transcription errors.

### Specimen processing and analysis

Whole-blood specimens were collected using cfDNA collection tubes (Streck, Omaha, NE). Automated cfDNA extraction was performed on fresh or frozen plasma using MyOne Dynabeads (Thermofisher Scientific, Waltham, MA). As previously described, sequencing libraries were created^11^ and multiplexed, clustered, and sequenced on HiSeq 4000 (Illumina, San Diego, CA).^5^ Sequencing data were normalized and analyzed using a novel algorithm to detect trisomies and subchromosomal, genome-wide CNVs ≥7 Mb in size as well as select microdeletions <7 Mb in size associated with 1p36 deletion, Wolf–Hirschhorn, Cri-du-chat, Langer–Giedion, Jacobsen, Prader–Willi, Angelman, and DiGeorge syndromes.^5,6^ Fetal fraction for each specimen was estimated as described by Kim et al.^12^ Samples without sufficient fetal fraction for a reliable result were considered nonreportable due to quantity not sufficient (QNS). Samples that failed to meet other laboratory quality metrics were considered nonreportable for technical reasons. Sample specific fetal fraction thresholds utilizing signal to noise ratio were used to determine if each sample was reportable, as previously described^13^ beginning in September 2016. Laboratory directors reviewed sequencing data from each sample before releasing results; directors had access to clinical information on the TRF when needed and available. Post-test genetic counseling was available to any patient with a positive result.

Turnaround time (TAT) is the in-lab time beginning when the specimen is received at the testing facility to the time a report is issued (in calendar days).

### Outcome collection

Collection of outcomes was approved by AspireIRB under clinical protocol SCMM-RND-402. Diagnostic outcomes were collected from two sources. First, screen-positive results were called out by a board-certified genetic counselor (GC) who requested information about future diagnostic testing. GCs tracked diagnostic testing and outcome when available from the ordering clinician (*n* = 1,236). Additionally, any ad hoc feedback provided on screen-negative cases was documented when provided to the laboratory.

A second subset of unique cfDNA cases (*n* = 419) were matched to corresponding diagnostic testing results (i.e., karyotype and/or single-nucleotide polymorphism [SNP] microarray results from chorionic villi, amniotic fluid, products of conception, or postnatal blood) from the internal commercial diagnostic testing laboratory (LabCorp/Integrated Genetics). Samples were matched based on unique patient identifiers (name and date of birth). Cell-free DNA and diagnostic samples collected within 90 days of each other were presumed to be from the same pregnancy. For samples collected more than 90 days apart but less than 200 days apart, clinical details were reviewed to determine whether the samples were indeed matched, and only those with consistent gestational ages and clinical details were considered matches (*n* = 7). For details regarding cytogenetic and microarray testing methodologies, see Supplementary materials.

An outcome was designated as true positive if the cfDNA finding was confirmed by diagnostic testing in the fetus, mother, or placenta. An outcome was designated as false positive when diagnostic testing on the placenta, fetus, or mother did not confirm the results from the cfDNA test, and as false negative when an event detectable by the assay was found on diagnostic testing, but not on the cfDNA test. True negatives were those cases with a negative cfDNA result and a normal diagnostic test. In most cases with multiple findings, each finding was counted individually (for example, a case that was a true positive for trisomy 21 and a false positive monosomy X were treated separately for calculating performance metrics for those results). However, five samples had two positive findings >7 Mb in which only one of the two events were confirmed; these samples were treated as partially concordant and counted as a true positive for calculating performance metrics. For details on the cases with complex categorizations, please see the Supplementary materials.

### Indications for testing

Indications for testing were provided by the ordering clinician on the TRF. Reasons for testing included the typical “high-risk” indications for aneuploidy: advanced maternal age (AMA), abnormal serum biochemical screening (SBS), ultrasound finding (USF), personal or family history of a chromosome abnormality (HX), other high-risk indication (HR-NOS), as well as those with no known high-risk indication (NO-HR). Multiple indications included those cases with two or more high-risk indications.

Of note, indication for testing is not a required field on the TRF. To resolve cases with no known indication, if maternal age was over 35, that sample was reassigned as AMA. Cases with NO-HR were then cross-referenced with the billing ICD-10 code provided to the lab and those with an ICD-10 code associated with AMA, HX, USF, or SBS were reassigned to the appropriate categories. Some ICD-10 codes suggested the patient was at increased risk for a chromosome abnormality but did not allow further assignment into one of the defined high-risk categories and were assigned to HR-NOS. Ultimately, 397 samples were reassigned from NO-HR to one of the high-risk categories after this process. NO-HR included both samples specifically designated as average risk on the TRF as well as samples with no provided indication that was unresolved by maternal age or ICD-10 diagnosis codes.

### Statistical analysis

Study data was statistically described using counts, rates, and measures of central tendency. A Cochran–Armitage trend using R.3.5 and library (DescTools) for testing trend in proportions was used to compare the trend of positive results among the three testing years. The trend of reason for referral was also similarly compared among the three testing years. A *p* value of <0.05 was considered statistically significant for inferential tests. A two by two contingency table was used to calculate sensitivity, specificity, and positive predictive value (PPV) using VassarStats (www.vassarstats.net). Positivity rates were calculated based on reportable samples.

## RESULTS

### Demographic details

During the study period, 55,517 samples were submitted for clinical testing via genome-wide cfDNA analysis at a single commercial laboratory. The average maternal age was 34 years (range 14–59) and stayed consistent year over year during the study period. The average gestational age was 14.8 weeks and again stayed relatively consistent year over year. Average maternal weight was 160.3 pounds and average turnaround time was 5.4 calendar days. Approximately 24.8% of samples were from providers outside of the United States.

Testing on amniotic fluid was the most common diagnostic sample type (56.7%), with “karyotype only” being the most common test ordered (58.4%). Testing on postnatal samples (13.1%), chorionic villi (12.5%), products of conception (6.3%), and multiple sample types (6.6%) were the next most common sample types. Fewer samples had testing on maternal blood (2.5%), with a small subset having testing on “unspecified” or “other” sample type (2.3%). “Microarray only” was ordered in 10.8% of cases, multiple tests were performed on 12.5% of samples, with “other” or “unspecified” diagnostic tests performed in 18.3%. Of note, 2.05% of cases had uniparental disomy (UPD) studies, but always in conjunction with another test (karyotype or microarray) and thus are represented in the “multiple tests” category. Figures S1, S2 in the Supplementary materials show the breakdown of the specimen type and type of testing ordered in the cases with confirmatory testing. Figures S3, S4 show the distribution of specimen type within the true positive and false positive cases. Differences are observed in the percent of cases with testing on amniotic fluid (41.6% of true positive cases vs. 66.5% of false positive cases), chorionic villi (15.8% vs. 6.6%), and products of conception (8.7% vs. 3.1%).

Diagnostic testing results were available in 42.5% (*n* = 1,142) of screen-positive samples, and 0.82% of screen-negative samples had diagnostic testing results available, with overall 2.98% of samples with diagnostic outcomes.

### Laboratory metrics

Maternal age was the most common indication for testing, both overall and in every clinical year of testing (Table 1). In year 1, the category “ultrasound findings” was the second commonest indication, but by year 3, this was surpassed by “no known high-risk indication.” “Multiple indications” remained relatively stable as the third most common category year over year. All indications, except maternal age, showed significant trends over time (*p* < 0.05). Table S2 in the Supplementary materials showed the *p* values and *Z*-scores for each indication.

**Table 1:** Indication for testing, overall and by testing year.

Indication	Year 1	Year 2	Year 3	Overall	Positivity rate
Maternal age	52.9%	54.1%	52.5%	**52.9%**	3.9%
Ultrasound findings	22.0%	16.7%	12.0%	**16.7%**	12.5%
No known high-risk indication	3.0%	7.2%	16.6%	**9.0%**	2.3%
Multiple indications	7.9%	8.1%	6.3%	**7.8%**	9.1%
Personal/family history	5.8%	5.2%	3.9%	**4.9%**	3.1%
Serum biochemical screening	5.7%	4.6%	4.0%	**4.7%**	4.6%
Other high-risk indication	2.9%	4.1%	4.8%	**4.0%**	2.2%

The right-most column shows the positivity rate for each indication.

### Test results

**Fig. 1:**
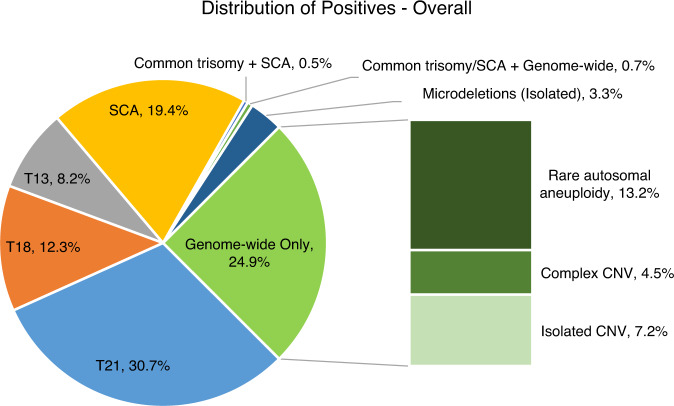
Distribution of positive results in the overall cohort of 55,517 samples. Common trisomy is a trisomy of chromosome 21, 18, or 13. Complex copy-number variant (CNV) includes cases with multiple events that involve at least one genome-wide event or events with CNV/aneuploidy. Mircrodeletions refers to the select list of microdeletions <7 Mb as described in “Materials and Methods.” Rare autosomal aneuploidy refers to aneuploidy of any chromosome excluding trisomies of 21, 18, or 13 and sex chromosome aneuploidies.

**Fig. 2:**
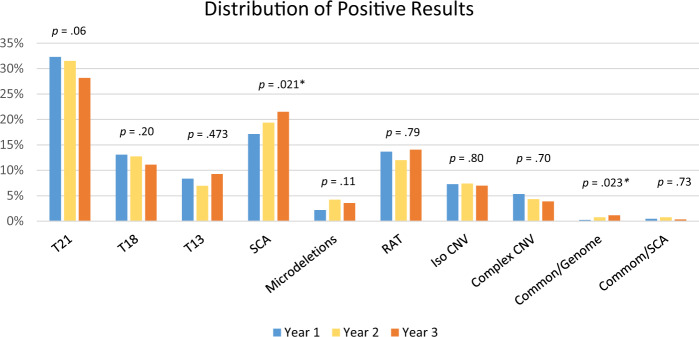
Graphic depicting the distribution of positives by year. Rare autosomal trisomies (RAT) also include two cases that were monosomies of autosomes. Microdeletions refer to the select list of microdeletions <7 Mb as described in “Materials and Methods.” Common/Genome refers to cases positive for a common trisomy and a genome-wide event, while Common/SCA refers to cases positive for a common trisomy and a sex chromosome aneuploidy. Categories with an asterisk (*) show a significant trend, although given the small sample size of the Common/Genome category, significance should be interpreted with caution. Corresponding *Z*-scores can be found in Table S3. *CNV* copy-number variant.

The overall positivity rate was 5.06% and shifted slightly over each of the cohort years (5.35% for year 1, 4.80% for year 2, and 5.09% for year 3, with year 1 being significantly different from years 2 and 3). The breakdown of the type of positive results stayed relatively consistent year over year. Figure 1 shows the overall breakdown of positive results, including details about the types of genome-wide only results. Approximately 25% of screen-positive results were considered “genome-wide” findings, representing rare trisomies and subchromosomal CNVs; this was the second most common screen-positive result. Figure 2 shows the year-over-year trends in positive cases, with the relative contribution from each type of positive result staying consistent, with the exception of the SCA and Common/Genome categories, with the latter having a small sample size. As expected, the majority of abnormalities identified were related to the common trisomies and SCAs. Yet, both overall and year over year, one of every four positive results would not have been identified by a “traditional” cfDNA assay.

There were 53,099 reportable samples; 4.35% of samples resulted in a nonreportable, either due to low fetal fraction (2.54%, quantity not sufficient/QNS) or technical failures (1.81%) related to laboratory quality metrics. Repeat specimens were received for 41.6% of the nonreportable cases, with a 70.5% success rate on redrawn specimens.

There were 2,687 samples with at least one positive finding. Of those, 2,531 samples had a single positive, while 155 samples (5.8%) had 2 or more positive findings. Performance metrics were calculated based on cases where diagnostic testing results were available (*n* = 1,569) and are provided in Table 2. For the genome-wide performance, the sensitivity, specificity, and PPVs for subchromosomal CNVs were 94.1%, 96.7%, and 72.6%, respectively, while the RATs showed 87.2% sensitivity, 90.7% specificity, and 22.4% PPV. Details for the number of true positives, true negatives, false positives, and false negatives used to calculate performance metrics can be found in Table S1 of the Supplementary materials. Of note, 39 true positive cases were confirmed only in the mother (most [*n* = 32] did not have fetal testing, while others [*n* = 7] had normal results from fetal testing) and another 8 true positive cases were confirmed in both the mother and fetus.

**Table 2:** Performance metrics for the cohort with diagnostic testing.

Condition (# positive with diagnostics)	Sensitivity (95% CI)	Specificity (95% CI)	PPV (95% CI)
T21 (*n* = 327)	99.4% (97.5–99.9%)	99.0% (98.3–99.5%)	96.3% (93.5–98.0%)
T18 (*n* = 121)	95.8% (90.0–98.4%)	99.5% (99.0–99.8%)	94.2% (88.0–97.4%)
T13 (*n* = 96)	98.7% (91.7–99.9%)	98.5% (97.7–99.0%)	76.0% (66.0–83.9%)
Monosomy X (*n* = 123)	95.8% (87.3–98.9%)	96.3% (95.2–97.2%)	55.3% (46.1–64.2%)
XXX (*n* = 24)	100% (77.1–100%)	99.6% (99.0–99.8%)	70.8% (48.8–86.6%)
XXY (*n* = 24)	92.0% (72.5–98.6%)	99.9% (99.6–100%)	95.8% (76.9–99.8%)
XYY (*n* = 8)	88.9% (50.7–99.4%)	100% (99.7–100%)	100% (59.8–100%)
Other SCA^a^ (*n* = 14)	84.6% (53.7–97.3%)	99.8% (99.4–100%)	78.6% (48.8–94.3%)
22q (*n* = 39)	88.4% (74.1–95.6%)	99.9% (99.6–100%)	97.4% (84.9–99.9%)
1p36 (*n* = 7)	100% (56.1–100%)	100% (99.7–100%)	100% (56.1–100%)
15q (*n* = 8)	100% (59.8–100%)	100% (99.7–100%)	100% (59.8–100%)
4p (*n* = 9)	100% (62.9–100%)	100% (99.7–100%)	100% (62.9–100%)
5p (*n* = 8)	100% (51.7–100%)	99.9% (99.5–100%)	75.0% (35.6–95.5%)
11q (*n* = 4)	100% (46.3–100%)	100% (99.7–100%)	100% (46.3–100%)
8q (*n* = 2)	100% (19.8–100%)	100% (99.7–100%)	100% (19.8–100%)
RAT^b^ (*n* = 183)	87.2% (73.6–94.7%)	90.7% (89.1–92.1%)	22.4% (16.7–29.3%)
>7 MB (*n* = 175)	94.1% (88.3–97.2%)	96.7% (95.6–97.5%)	72.6% (65.2–78.9%)

Of note, if a sample had multiple positive findings of the same type reported, that sample was counted a single time and was treated as a single true positive if at least one of the findings was confirmed and as a single false positive if none of the findings were confirmed. In cases with multiple findings of different types, those findings are counted individually in the table, resulting in some overlap between categories. Sensitivity  =  true positives/(true positives  +  false negatives); specificity  =  true negatives/(true negatives  +  false positives); PPV  =  true positives/all positives.

*CI* confidence interval, *PPV* positive predictive value, *RAT* rare autosomal trisomy, *SCA* sex chromosome aneuploidy.

^a^Other SCA includes events involving the sex chromosomes that do not fit in one of the other defined SCA conditions, such as polysomy and copy-number variants (CNVs) involving a sex chromosome.

^b^RAT also includes two cases of monosomies of autosomes.

## DISCUSSION

### Demographic and utilization trends

This study details three years of genome-wide cfDNA screening from one laboratory. While the majority of abnormalities identified were common trisomies and SCAs, one of every four positive cases would not have been identified with a “traditional” cfDNA assay, similar to the historical literature on potentially undetected chromosome abnormalities on cfDNA as compared with karyotype.^3,4^ Genome-wide findings were second only to trisomy 21 in frequency, as seen in Fig. 1. This was a trend that stayed consistent year over year, even as the indications for testing shifted. The consistency of the genome-wide contributions to the positive findings, over time and changing referral indications, is noteworthy and deserves further study.

In the first testing year, approximately 1 in 5 patients opted for testing due to ultrasound findings, yet by testing year 3, this dropped to approximately 1 in 8.5 patients. This appears to correspond to an increased proportion of patients with no known high-risk indication. Although limited in the ability to determine whether these are truly ‘”average risk” patients, these data suggest that providers may find value in screening a broader population of patients for genome-wide events, especially given that the risk for a CNV is not associated with maternal age. For patients without a high-risk indication, the risk for a CNV detectable by this assay is greater than the risk for T18 or T13.^14^ This raises the question of whether this testing should indeed be offered to patients without the typical “high-risk” indications. Other maternal demographic characteristics (maternal age, gestational age, weight) also stayed relatively consistent year over year.

### Test performance

Test performance for common aneuploidies and SCAs is similar to those previously reported on genome-wide cfDNA tests,^5,15–17^ yet data on test performance for other genome-wide events were previously limited. Although the validation study showed high sensitivity and specificity across the genome,^5^ the number of affected cases was a limitation. While overall outcomes are limited, especially in negative cases, a strength of the current study is diagnostic testing outcomes in over 40% of positive cases, allowing reasonable confidence in some aspects of the performance calculations, especially PPV.

Genome-wide CNVs, including the select microdeletions, showed high sensitivities, specificities, and PPVs with >70% of positive results confirmed by diagnostic testing. Unlike targeted assays, this genome-wide assay avoids the cumulative false positive rates due to multiple-hypothesis testing, instead using the same circular binary segmentation (CBS) approach used for microarray detection of CNVs as previously described.^6^ Although the test performance reflected in this clinical cohort is slightly lower than the clinical validation,^5^ this study reflects a diverse population of clinical samples, including samples with known maternal events, mosaicism, or co-twin demise. Furthermore, potential biases on which cases in this cohort had diagnostic outcomes available may skew test performance calculations, as discussed in “Limitations.” A PPV of >72% for subchromosomal CNVs is consistent with the modeled PPV predicted in the clinical validation study and is higher than seen for CNVs in other studies.^5,7,16–18^

Although the PPV for RATs (22.4%) was lower than for subchromosomal events (CNVs), the PPV is still higher than that of serum screening for common aneuploidies.^19^ RATs may be present in the fetus as either a full or mosaic event or as UPD resulting from a presumed trisomic rescue. The PPV is similar to or slightly higher than seen in studies looking at RATs in cfDNA, although the patient populations in some of those studies differ from this cohort.^7,15–17^ Regardless of whether a RAT is confirmed by diagnostic testing, there remains the potential for clinical relevance to the pregnancy. A recent presentation reviewed a subset from this current cohort, (cases screening positive for an isolated rare aneuploidy) and suggests that nearly 47% of cases that screened positive for a rare aneuploidy were either confirmed in the fetus/placenta and/or resulted in an adverse outcome, defined as growth restriction, preterm labor/delivery, miscarriage/fetal demise, or structural ultrasound anomalies.^20^ Other studies have also shown a high rate of adverse outcomes when RATs are seen by cfDNA.^17,21^ Further data are needed to precisely quantify these risks and make subsequent recommendations for pregnancy management.

Monosomies, beyond those of the X chromosome, are rare with an incidence of ~1/25,000 in this cohort and were grouped with rare autosomal trisomies for analysis. While rare, these are reported within the parameters of the testing as there is the possibility of a cosegregating trisomic cell line or rescue event with subsequent UPD or mosaicism.^22–25^

The nonreportable rate for this genome-wide assay (4.35%) is higher than that of the laboratory assay for common aneuploidies (0.9%),^25,26^ because reliably calling genome-wide CNVs requires more robust and cleaner sequencing data than calling of traditional aneuploidies. This is reflected in a higher fetal fraction requirement and signal to noise ratio requirement for this genome-wide assay compared with the traditional cfDNA assay for common aneuploidies.^14^ In part, this challenge is overcome by increasing the number of sequencing reads per sample. More than half of samples receiving a nonreportable result did not submit a repeat specimen. This may be related to society guidelines which recommend consideration of diagnostic testing after a nonreportable result.^8,9^ Diagnostic testing results were unavailable for nearly all (>95%) cases with a nonreportable result, limiting the ability to draw any conclusions about risk for aneuploidy in the cohort receiving a nonreportable result.

### Limitations

A significant limitation for this study is the population, as the patients in the overall study cohort were mostly women with a high-risk indication. Additionally, the cohort with diagnostic testing may be biased simply by the fact that they proceeded with additional testing and may be different from the population of patients who did not proceed with diagnostic testing. In addition, we do not have visibility to cases where diagnostic testing was performed in a different laboratory, except when this information was provided to the laboratory by the clinician during the outcome follow-up process as described. Overall, 2.98% of specimens had diagnostic testing results available; however, when studying only positive cases, over 42% had diagnostic results. Adjudication of the testing indications (reassigning patients over 35 years old to AMA, utilization of ICD-10 codes to reassign NO-HR patients) may have introduced additional bias. However, only a small subset (*n* = 397) representing less than 1% of all cases were reassigned using maternal age or ICD-10 resolution.

Finally, this is a retrospective study of data available to a clinical laboratory and thus may reflect biases in the cases and types of data available. As noted earlier, outcome was collected by two methods: feedback from clinicians and matching of cfDNA results with diagnostic specimens submitted to the same laboratory. Clinicians may be more likely to report a discordant outcome than a concordant outcome, with true negatives being least likely to warrant follow-up communication. These patients may also be less likely to have diagnostic testing. Conclusions regarding specificity are limited given the relatively small number of euploid cases with diagnostic testing available in this study; negative predictive values were not calculated for this reason. Ideally, complete diagnostic information would be available for every case, including placental testing and UPD studies, but this is unrealistic for a retrospective study of this size. Cases had variable types of diagnostic testing and specimen types. Given these limitations and considering the biases of the cohort with diagnostic testing, the results may not be generalizable to a general obstetric population.

Discordance between cfDNA screening and diagnostic results is an expected occurrence due to a variety of biological factors. One such limitation is presence of a co-twin demise. In the cohort with diagnostic testing, 11 false positive cases had a known co-twin demise. Other cases with “weaker” aneuploidy signals by cfDNA may be discordant due to unrecognized co-twin demise or low level, cryptic mosaicism.

Mosaicism is another recognized limitation of cfDNA screening and may result in discordant results as both false positives related to confined placental mosaicism (CPM types I and III) and false negatives related to mosaicism confined to the mesenchymal layer of the placenta (CPM type II) or to the fetus (CPM type V). Efforts have been made to try to account for this phenomenon, where possible. Mosaicism ratio is calculated by dividing the fraction estimated for the detected event (aneuploid chromosome or chromosomal segment) by the fetal fraction estimated across all chromosomes and may be useful in predicting which samples might have a lower positive predictive value as previously described.^10,27,28^ When mosaicism is suspected, amniocentesis may be preferable to chorionic villus sampling to determine fetal status. Of the false negative cases, 26.5% (*n* = 9) could be attributed to documented mosaicism on diagnostic testing, especially with SCAs and RATs. Two of those cases involved mosaicism of monosomy X with either an XY or XX cosegregating cell line on amniocentesis, which may result in essentially normal cfDNA data depending on the mosaic load in the placental trophoblast. For six false negative RATs, it is noteworthy that four of these cases showed mosaicism by diagnostic testing, with at least two showing very low level mosaicism, at or below ~10%. Due to this residual risk for very low level mosaicism, especially given laboratory thresholds of ~10–20%, which may not be reportable on diagnostic testing, the sensitivity for RATs should be treated as an estimate.

### Future directions and conclusion

Professional societies recommend that diagnostic testing with karyotype or microarray should be made available to all patients, regardless of age or other risk factors.^8,9,29^ However, some women will initially decline diagnostic testing, and for others, access to diagnostic testing may be limited by gestational age, proximity to a qualified physician, patient aversion to diagnostic testing, or other factors. In these situations, screening may be a more acceptable option. For those patients, genome-wide cfDNA screening provides more clinically relevant information than was previously available from serum screening or cfDNA testing limited to the common trisomies. Given that professional societies desire more performance data on cfDNA,^8,9^ this study and others in the future aim to provide the information needed for patients and providers to make informed choices regarding prenatal screening. Additionally, ensuring that patients have access to nondirective, pretest counseling on the different aneuploidy testing options remains imperative, especially as available testing increases in complexity.

The clinical utility of expanded content in the average risk screening population requires continued prospective study. While early data from the second phase of the TRIDENT study are promising in this area,^7^ additional evidence from this group and others will be helpful in providing comprehensive screening options and management protocols for patients.

### Supplementary information

Supplementary Information

## Data Availability

Data that support the findings of this study are available upon request.
