# Correction to: Three years of clinical experience with a genome-wide cfDNA screening test for aneuploidies and copy number variants

**DOI:** 10.1038/s41436-021-01190-1

**Published:** 2021-05-06

**Authors:** Erica Soster, Theresa Boomer, Susan Hicks, Samantha Caldwell, Brittany Dyr, Jason Chibuk, Eyad Almasri

**Affiliations:** Integrated Genetics, San Diego, CA USA

Correction to: *Genetics in Medicine* 2021; 10.1038/s41436-021-01135-8; published online 17 March 2021

Unfortunately, an error occured in Fig. 2. The corrected Fig. 2 is given below.

**Fig. 2:**
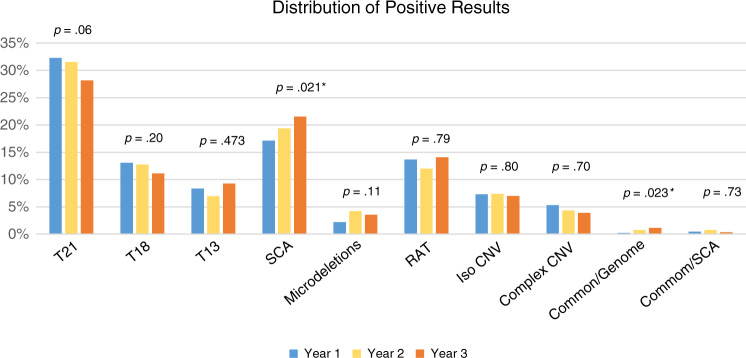
Graphic depicting the distribution of positives by year. Rare autosomal trisomies (RAT) also include two cases that were monosomies of autosomes. Microdeletions refer to the select list of microdeletions <7 Mb as described in “Materials and Methods”. Common/Genome refers to cases positive for a common trisomy and a genome-wide event, while Common/SCA refers to cases positive for a common trisomy and a sex chromosome aneuploidy. Categories with an asterisk (*) show a significant trend, although given the small sample size of the Common/Genome category, significance should be interpreted with caution. Corresponding Z-scores can be found in Table S3. CNV copy-number variant.

The original article has been corrected.

