# Supplementary material for: Three years of clinical experience with a genome-wide cfDNA screening test for aneuploidies and copy-number variants
Source: Genet Med. 2021 Mar 17;23(7):1349–55. doi: 10.1038/s41436-021-01135-8 (PMC8257487; doi:10.1038/s41436-021-01135-8)
Supplement: Supplementary file 1 — Supplementary Information [file 41436_2021_1135_MOESM1_ESM.pdf]

Supplementary Materials for Soster et al

Diagnostic testing at LabCorp and Integrated Genetics

Amniotic fluid and chorionic villus specimens were cultured, harvested, and G-banded according to standard methods. For *in situ* amniocyte analysis, at least 15 primary colonies from two or more independent cultures were examined. If fewer than 15 primary colonies were available, a total of 20 cells from both primary and trypsinized cultures were examined. For chorionic villus specimens, at least 20 metaphase cells from two or more independent cultures were examined. In cases of mosaicism, abnormal cell lines were present in at least two independent cultures. For products of conception, at least 20 metaphase cells were examined.

All chromosomal microarray studies were performed utilizing the ThermoFisher® Cytoscan® HD array [ThermoFisher ® and CytoScan® are Registered Trademarks of ThermoFisher, Inc.]. This array contains approximately 2.695 million markers across the entire human genome. There are approximately 743,000 single nucleotide polymorphic probes (SNPs) and 1,953,000 structural non-polymorphic probes. On the average there is approximately 0.88 kb between each marker. DNA was extracted utilizing standard methods and 250ng of total genomic DNA extracted was digested with Nsp1 and then ligated to Nsp1 adaptors, and amplified using Titanium Taq with a GeneAmp PCR System 9700. PCR products were purified using AMPure beads and quantified using NanoDrop 8000. Purified DNA was fragmented and biotin labeled and hybridized to the ThermoFisher Cytoscan® HD GeneChip. Data was analyzed using Chromosome Analysis Suite. The analysis is based on the GRCh37/hg19 assembly.

Details for samples with complex outcome categorization

The 36 false negative events resulted from a total of 34 samples. One sample was a false negative for T18 and XXY while another sample was a false negative for two events >7 Mb.

13 samples were counted in more than one category in the table:

- One sample was false negative and false positive (false negative T18, false positive monosomy X)
- Two samples were both a true positive and a false negative
    - TP T18 and FN mosaic monosomy X/XXX (correctly identified trisomy 18 but did not identify mosaicism for monosomy X/XXX in the sample)
    - TP 11q and FN >7 Mb
- Twelve samples were both true positives and false positives (for separate events like T21 and monosomy X) (Accounts for 12 TP and 12 FP)
    - TP T21 with FP monosomy X
    - TP T18 with FP monosomy X
    - TP >7 Mb with FP monosomy X
    - TP T18 and FP RAT
    - TP 15q and FP RAT
    - 3 cases TP T21 and FP RAT
    - 3 cases TP 11q and FP >7 Mb event
    - 1 case TP 1p36 and FP >7 Mb event
- Another 5 samples were partially concordant and treated as true positives
    - 5 cases 2x >7 Mb events (only one >7 Mb event confirmed)
- 2 cases with true positive for one event and unknown diagnostic results for second event
    - 2 cases TP T21 and unknown for >7 Mb

	# reported positive (incidence in reportable cohort)	# positive results with diagnostic results (% positives with diagnostic results)	# reported negative with diagnostic results	TP	FP	TN	FN
**T21**	841 (1.58%)	327 (38.9%)	1242	315	12	1240	2
**T18**	341 (0.64%)	121 (35.6%)	1447	114	7	1442	5
**T13**	221 (0.42%)	96 (43.4%)	1473	73	23	1472	1
**MX***	382 (0.72%)	123 (32.1%)	1446	68	55	1443	3
**XXX**	64 (0.12%)	24 (37.5%)	1545	17	7	1545	0
**XXY**	63 (0.12%)	24 (38.1%)	1545	23	1	1543	2
**XYY**	25 (0.05%)	8 (32.0%)	1561	8	0	1560	1
**Other SCA**	22 (0.04%)	14 (63.6%)	1555	11	3	1553	2
**22q**	60 (0.11%)	39 (65.0%)	1514	38	1	1509	5
**1p36**	8 (0.02%)	7 (87.5%)	1562	7	0	1562	0
**15q**	13 (0.02%)	8 (61.5%)	1561	8	0	1561	0
**4p**	16 (0.03%)	9 (56.3%)	1560	9	0	1560	0
**5p**	14 (0.03%)	8 (57.1%)	1561	6	2	1561	0
**11q**	5 (0.01%)	5 (100%)	1564	5	0	1564	0
**8q**	2 (<0.01%)	2 (100%)	1567	2	0	1567	0
**RAT****	371 (0.70%)	183 (49.3%)	1386	41	142	1380	6
**>7 MB**	295 (0.56%)	175 (59.3%)	1394	127	48	1386	8

TP: True positives; FP: False positives: TN: True negatives; FN: False negatives

Table S1: Detailed results and outcomes for each event used to calculate observed performance metrics.

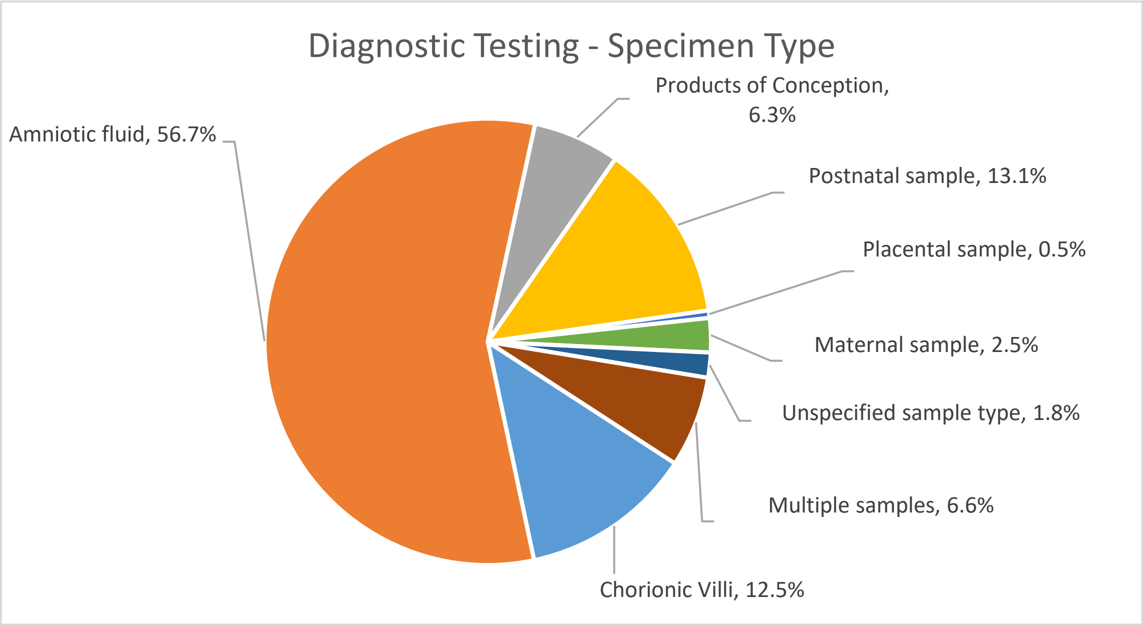

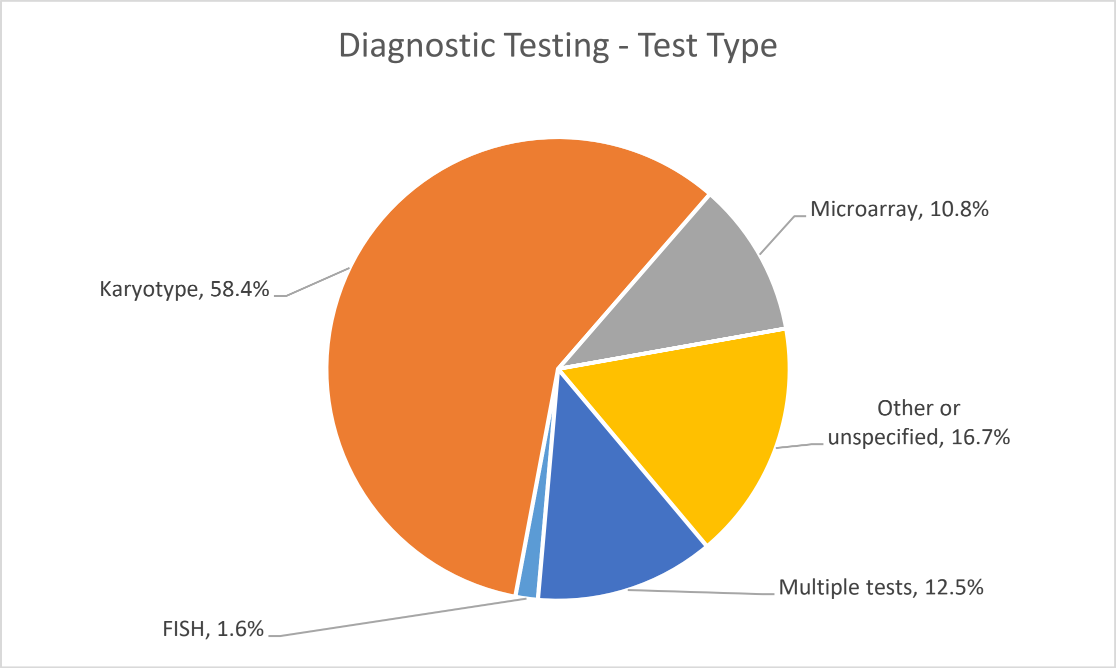

FIGURES S1 and S2: *A pie chart showing the breakdown of specimen type for diagnostic testing (S1) and assay test type ordered on diagnostic testing (S2).*

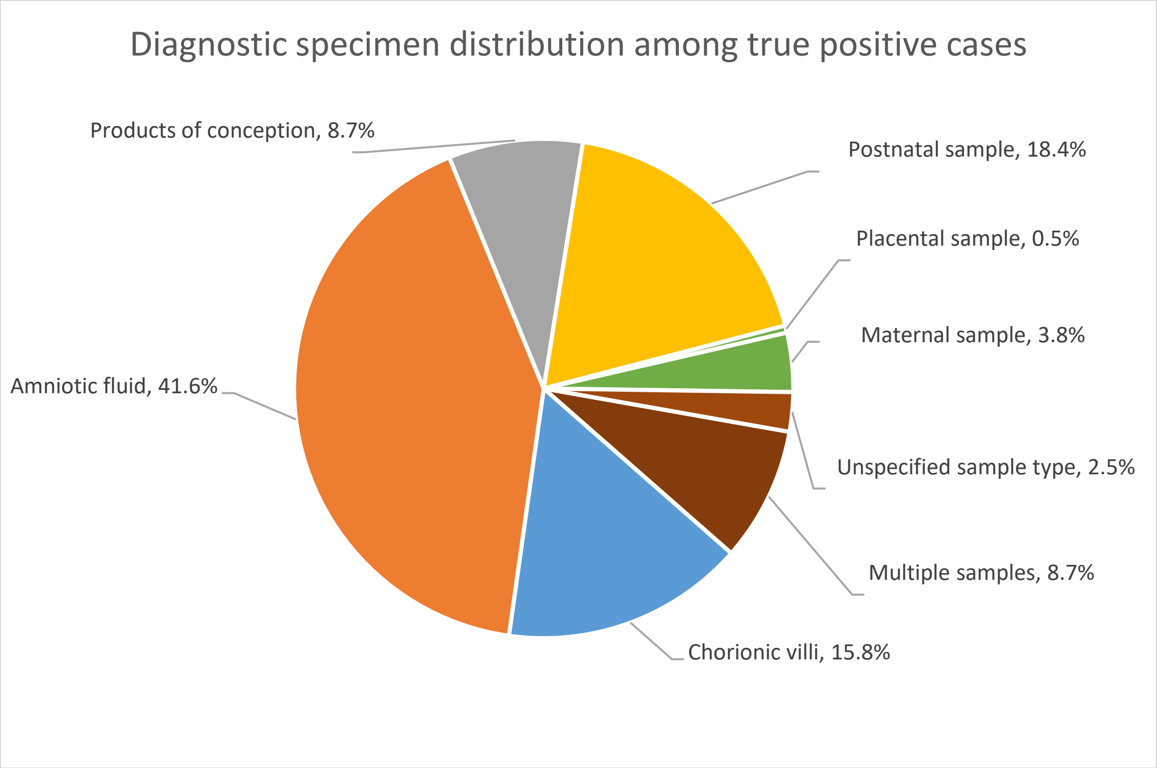

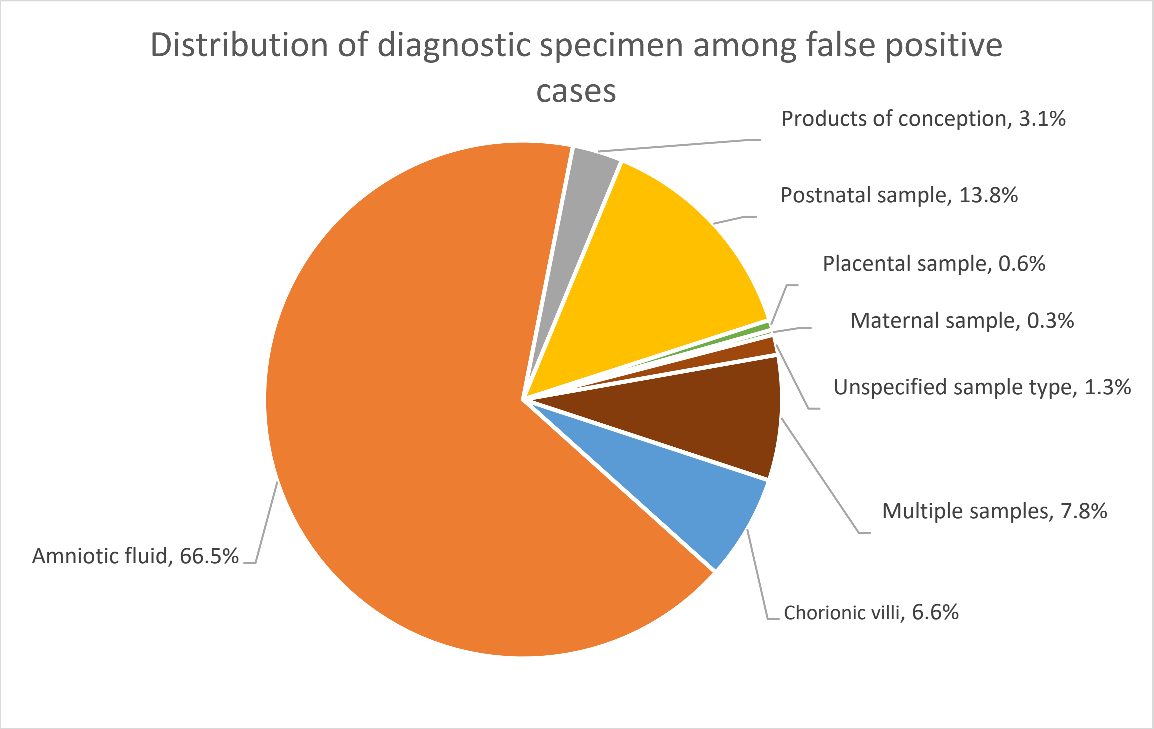

Figures S3 and S4: Two pie charts showing the breakdown of diagnostic testing specimen type among the true positive cases and false positive cases.

	P value	Z score	Significant trend?
**Maternal Age**	0.3802	-0.87748	No
**Ultrasound findings**	0	-24.95	Yes
**No high risk indication**	0	44.82	Yes
**Multiple indications**	6.00E-09	-5.8	Yes
**Personal/family history**	0	-8.1	Yes
**Serum screening**	0	-7.3306	Yes
**Other high-risk indication**	0	9.1146	Yes

Table S2: Associated p values and Z-scores for reason for referral. All of the trends are significant except maternal age.

	P value	Z score	Significant trend?
**Trisomy 21**	.06	-1.87	No
**Trisomy 18**	.20	-1.25	No
**Trisomy 13**	.473	0.71	No
**SCA**	.021	2.3	Yes
**Microdeletions**	.11	1.55	No
**RAT**	.79	0.26	No
**Isolated CNV**	.80	-0.25	No
**Complex CNV**	.70	-1.4	No
**Common/Genome**	.023	2.27	Yes
**Common/SCA**	.73	-0.35	No

Table S3: Associated p values and Z-scores for positive results by year. Only the SCA and Common/Genome categories show a significant trend, although the small sample size of the Common/Genome category should be interpreted with caution.
